# Emerging frontiers in glioma therapy: the evolving role of glioma vaccines

**DOI:** 10.3389/fimmu.2026.1781820

**Published:** 2026-05-11

**Authors:** Renfei Gao, Zhenyu Gong, Yue He, Yongluo Jiang, Hao Duan, Hongxiang Wang

**Affiliations:** 1 Department of Neurosurgery, Shanghai Changhai Hospital, Naval Medical University, Shanghai, China; 2 State Key Laboratory of Vaccines for Infectious Diseases, Department of Laboratory Medicine, National Innovation Platform for Industry-Education Intergration in Vaccine Research, School of Public Health, Xiamen University, Xiamen, China; 3 Xiang An Biomedicine Laboratory, Department of Laboratory Medicine, National Innovation Platform for Industry-Education Intergration in Vaccine Research, School of Public Health, Xiamen University, Xiamen, China; 4 Department of Neurosurgery/Neuro-Oncology, State Key Laboratory of Oncology in South China, Guangdong Provincial Clinical Research Center for Cancer, Sun Yat-Sen University Cancer Center, Guangzhou, China; 5 State Key Laboratory of Oncology in South China, Collaborative Innovation Center for Cancer Medicine, Guangdong Provincial Clinical Research Center for Cancer, Sun Yat-sen University Cancer Center, Department of Nuclear Medicine, Sun Yat-sen University Cancer Center, Guangzhou, China; 6 Guangdong Key Laboratory of Nasopharyngeal Carcinoma Diagnosis and Therapy, Collaborative Innovation Center for Cancer Medicine, Guangdong Provincial Clinical Research Center for Cancer, Sun Yat-sen University Cancer Center, Department of Nuclear Medicine, Sun Yat-sen University Cancer Center, Guangzhou, China

**Keywords:** glioblastoma, glioma, glioma vaccines, immune checkpoint inhibitors, immunotherapy, tumor microenvironment

## Abstract

Glioma, particularly glioblastoma (GBM), remains one of the most lethal primary brain tumors, characterized by aggressive biological behavior, profound intratumoral heterogeneity, and a highly immunosuppressive tumor microenvironment. Despite encouraging advances in cancer immunotherapy, durable clinical benefit from glioma vaccines has remained limited, highlighting a substantial translational gap between early immunogenicity and long-term therapeutic efficacy. In this review, we provide an integrative overview of current glioma vaccine strategies, including peptide-based, dendritic cell–based, nucleic acid–based, and autologous tumor-derived platforms, with a particular focus on their biological rationale, clinical performance, and translational limitations. Rather than merely summarizing recent developments, we critically examine the shared factors that have constrained clinical success, including antigen heterogeneity, immune escape, HLA restriction, treatment-related immunosuppression, and the inhibitory effects of the glioma tumor microenvironment. We further discuss how these challenges have shaped the emerging rationale for combination strategies involving vaccines with chemotherapy, anti-angiogenic agents, immune checkpoint blockade, and other immunotherapeutic modalities. Finally, we highlight forward-looking design principles for next-generation glioma vaccines, including improved antigen selection, biomarker-guided patient stratification, microenvironment-aware therapeutic design, and more predictive translational models. Overall, we propose that future progress in glioma vaccine therapy will depend not only on improving vaccine platforms themselves, but also on integrating them into biologically rational and clinically informed therapeutic frameworks.

## 1 Introduction

**Figure 1:**
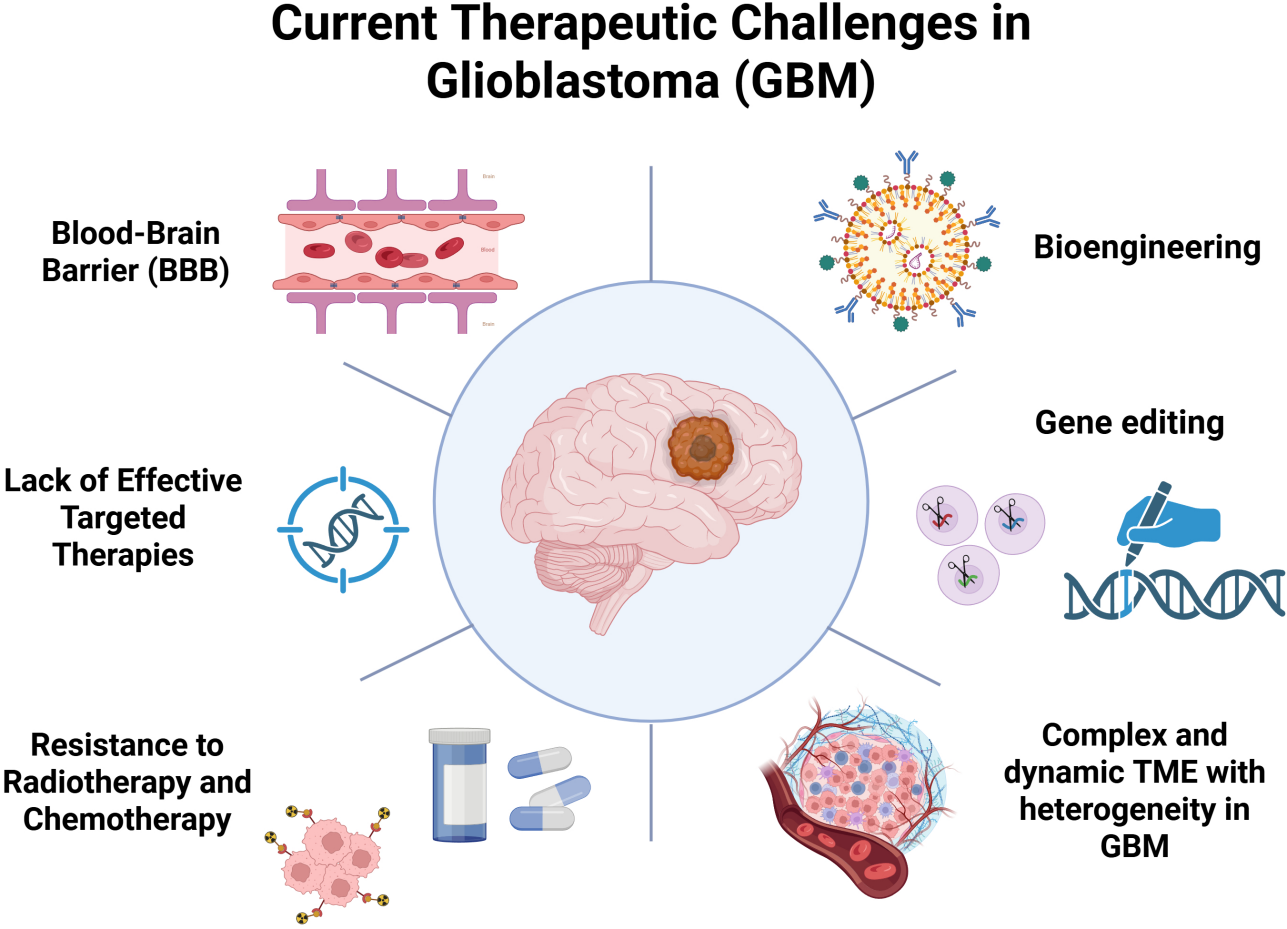
Current therapeutic challenges in glioblastoma (GBM). Due to its highly dynamic and complex microenvironment components and unique intratumoral heterogeneity, GBM is in urgent need of one or more combination therapies for precise target attacks. These therapies can be new bioengineering, exogenous editing methods, drugs and so on.

Gliomas are among the most common primary intracranial tumors and include astrocytomas, oligodendrogliomas, and ependymomas. Gliomas are categorized by the World Health Organization (WHO) into grades I–IV according to histological characteristics and clinical outcomes (1), and different grades have significant differences in the treatment and prognosis of brain glioma. Low-grade gliomas (LGG) of grades I and II often have a good prognosis and are mostly treated with local surgical excision in conjunction with radiation and chemotherapy (2). Grades III and IV are high-grade gliomas (HGG) requiring extensive surgical resection with adjuvant therapies, yet patients often experience poor outcomes (3). Glioblastoma (GBM), categorized as WHO grade IV *IDH*-wild-type (*IDH*wt) tumors (4), is the most prevalent, aggressive, and lethal primary malignant brain tumor of the central nervous system (CNS) in adults (5), accounting for 55% of glioma cases and carrying a 5-year survival rate below 5% (6). Firstly, its highly invasive malignant astrocytes frequently result in incomplete surgical resection (7). Secondly, GBM is characterized by a highly immunosuppressive microenvironment that limits the invasion of anti-GBM immune cells and prevents the establishment of anti-GBM immunity. Furthermore, the blood-tumor barrier (BTB) and blood-brain barrier (BBB) in GBM act as physiological obstacles that hinder the effective delivery of treatments from the systemic circulation to GBM tissues (8). Despite four decades of research efforts, patient prognosis remains largely unchanged (9). As illustrated in **Figure 1**, this persistent therapeutic failure reflects the convergence of multiple barriers, including intratumoral heterogeneity, invasive growth, restricted drug delivery, and a profoundly immunosuppressive tumor microenvironment.

The current standard-of-care Stupp regimen for GBM combines maximal surgical tumor resection combined with chemotherapy, radiotherapy (RT) and targeted therapy, primarily using temozolomide (TMZ) (10, 11). Despite efforts, it remains primarily palliative, with adult patients typically surviving a median of 15 months after diagnosis (12, 13). This is because complete surgical resection is usually unachievable (14), the high recurrence rates of GBM are associated with glioblastoma stem cells (GSCs) and the tumor’s invasive nature. GBM is usually diagnosed only after clinical symptoms appear, by which point its invasive cells have often already spread into eloquent brain areas that handle sensory input, motor control and language coordination (15, 16), making total removal challenging. Complete oncological excision is still technically impossible in the majority of instances, despite the fact that contemporary intraoperative imaging techniques have enhanced tumor margin visibility (17).

Despite standard multimodal therapy, overall survival (OS) for patients with GBM remains dismal even with the standard and aggressive therapies like chemotherapy and radiotherapy (18). The median survival time for GBM is approximately 12.5–15 months, and 2-year and 5-year survival rates with standard treatment are 25% and 10%, respectively (19). Additionally, the tumor’s resistance, the BBB’s obstruction of therapeutic delivery, and an immunosuppressive microenvironment all contribute to treatment resistance, making the prognosis still bleak (17). Given this constraint, there is now a pressing need to identify more effective alternative treatments for GBM. Glioma vaccines have emerged as a rapidly advancing therapeutic strategy designed to harness the immune system’s innate ability to detect and eliminate tumor cells. Their mechanism is based on the presentation of tumor-associated antigens, which in turn stimulates a focused immune response against the tumor. The investigation of vaccines based on peptides, dendritic cells, tumor cells, and viral vectors has shown promise in enhancing glioma cell immune recognition and generating targeted, long-lasting immune responses. These developments highlight a pivotal shift toward incorporating immunotherapy into the management of glioma (17).

Encouraging preclinical and early clinical studies have supported continued interest in vaccine-based immunotherapy as a potential strategy for glioma treatment (20). These vaccines seek to trigger a specific anti-tumor immune response by exposing the immune system to tumor-associated antigens. This progress marks a significant step forward in advancing immunotherapy for glioma (17).

Despite encouraging immunogenicity and survival signals reported in multiple early-phase studies, glioma vaccines have not yet produced consistent and durable clinical benefit across the field. This translational gap likely reflects the combined effects of intratumoral antigen heterogeneity and temporal evolution of glioma, which enable immune escape under selective pressure; the limited antigen coverage and HLA restriction inherent to certain vaccine platforms; and the profoundly immunosuppressive tumor microenvironment characterized by T-cell exhaustion, regulatory immune cell infiltration, and impaired antigen presentation. In addition, treatment-related factors, such as corticosteroid use and the lymphodepleting effects of standard chemoradiotherapy, may further constrain vaccine-induced immune responses. Finally, limitations in clinical trial design, including small sample sizes, insufficient biomarker-based patient stratification, and the mismatch between immunogenicity endpoints and long-term clinical outcomes, also contribute to the difficulty in translating early signals into durable therapeutic benefit. Therefore, a critical evaluation of glioma vaccine strategies requires not only cataloguing existing platforms and trials but also identifying the shared biological and clinical principles that govern their success or failure.

Distinct from prior reviews that primarily focus on cataloguing vaccine platforms or summarizing clinical trial outcomes, this review aims to provide a more integrative and translationally oriented perspective on glioma vaccines. Specifically, we emphasize three key aspects: (1) a critical synthesis of the translational gap between early-phase immunogenicity and late-phase clinical efficacy, (2) a mechanistic integration of tumor evolution, immune escape, and microenvironmental constraints that shape vaccine response, and (3) a conceptual framework for rational combination strategies designed to overcome sequential barriers to effective antitumor immunity. By linking preclinical mechanisms with clinical outcomes and highlighting shared limitations across vaccine platforms, we aim to move beyond descriptive summaries and provide design-oriented insights for next-generation glioma vaccine development.

## 2 Development of glioma vaccines

**Figure 2:**
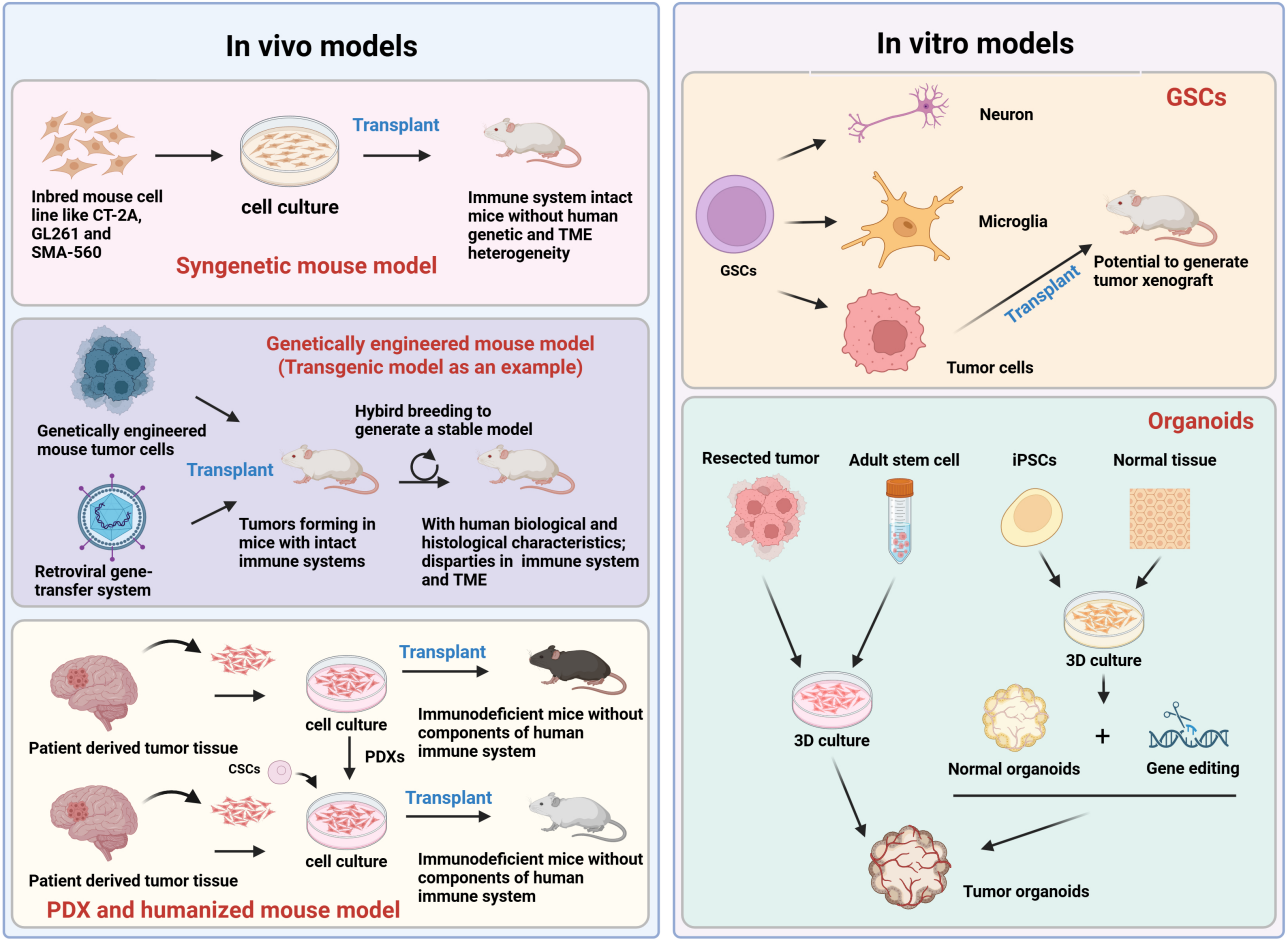
Commonly used preclinical models for glioma immunological research. The preclinical models can be categorized into *in vivo* and *in vitro* subsets. For glioma immunological research, *in vivo* models include syngeneic mouse models, GEMMs, patient-derived xenografting (PDX), and humanized mouse models. Syngeneic mouse models involve the transplantation of glioma cell lines such as CT-2A, GL261, SMA-560 into mice with an intact immune system. In GEMMs, genetically engineered mouse tumor cells are transplanted into immunocompetent mice via retroviral gene-transfer platform, then robust models are generated by cross-breeding the immune system intact mice. Both PDX and humanized mouse models are similar in that the tumor tissues are human-derived. A major discrepancy is that PDX and humanized models involve immunodeficient mice that do not express all components of the human immune system. Organoids can be derived from resected tumor tissues, adult stem cell, iPSCs, or normal tissues. CSCs, cancer stem cells; GEMM, genetically engineered mouse model; GSCs, glioma stem cells; iPSCs, induced pluripotent stem cells.

Although anti-glioma immunotherapies have shown considerable efficacy in rodent models, current preclinical systems remain limited in their ability to accurately recapitulate human immune responses and the heterogeneity of human glioma (21). **Figure 2** summarizes the major preclinical modeling platforms currently used in glioma immunology and highlights why more predictive translational models are urgently needed for vaccine development. Improved preclinical models are desperately needed to assess the anti-glioma effectiveness of immunotherapy, especially vaccinations, whether given singly or in combination (22).

### 2.1 Types of vaccines

Clinical trials are currently examining a variety of single or combined vaccination regimens, including peptide vaccines, dendritic cell (DC) vaccines, RNA/DNA vaccines, and autologous tumor-derived platforms (22). To facilitate comparison across these platforms, their key characteristics, including mechanisms, advantages, limitations, and clinical development stages, are summarized in **Table 1**. While DC vaccinations are loaded with tumor antigens, peptide and DNA vaccines deliver antigens specific to tumors. Viral vectors are used by mRNA vaccines to elicit strong immune responses (23, 24). Numerous randomized clinical trials have also demonstrated their clinical usefulness.

**Table 1:** Comparison of the mechanisms and advantages/disadvantages of the four main types of vaccines.

Vaccine platform	Mechanism of action	Advantages	Disadvantages	Clinical stage
Peptide-based	Uses specific TAA/Neoantigen epitopes to prime T-cells.	Easy to manufacture; high safety; cost-effective.	MHC restriction; high risk of immune evasion/antigen loss.	Phase II/III (e.g., SurVaxM).
DC-based	Professional APCs loaded with antigens to orchestrate adaptive immunity.	Broad immune response; targets "cold" tumors.	High cost; logistically complex.	Phase III (e.g., DCVax-L).
RNA/DNA	Delivers genetic sequences for endogenous antigen expression.	Fast production; no MHC restriction; strong CD8+ response.	Potential stability issues; delivery efficiency concerns.	Phase I/II.
Autologous Cell	Uses irradiated tumor cells to induce polyclonal response.	Targets multiple known and unknown TAAs.	Weaker anti-tumor activity in some recent trials.	Phase I/II.

#### 2.1.1 Tumor cell–derived vaccines

Autologous tumor cell vaccination boosts humoral and cell-mediated immunity against antigenic epitopes and induces a polyclonal immune response that targets several tumor-associated antigens (TAAs), including potentially undetectable ones (1). Patients with recurrent glioblastoma (rGBM) can be safely immunized with autologous tumor-derived peptides from GBM. Significant infiltration of localized CD_4_^+^, CD_8_^+^, CD_56_^+^, and IFN γ-producing T lymphocytes targeting autologous tumor-derived peptides bound to HSP-96 was seen in post-vaccination brain biopsies (25). In addition, employing tumor lysate vaccines and strong immune response modulators like imiquimod or poly-polyinosinic-polycytidilic acid (ICLC) adjuvants, the National Institutes of Health (NIH) Clinical Center has carried out a promising effort to promote cell-mediated immune responses (**Table 2**). However, despite the noted feasibility and safety of vaccination, recent clinical trials have shown that vaccination with irradiated autologous glioma exhibits relatively weak anti-tumor activity (1).

**Table 2:** Clinical trial of tumor lysate vaccine in glioma.

Tumor type	Patient cohort	Research content	Interventions	Status	Phase	Identifier
Recurrent HGG and DMG/​DIPG	24	Phase I Trial: CD200 Activation Receptor Ligand (CD200AR-L) and Allogeneic Tumor Lysate Vaccine Immunotherapy with Adjuvant Reirradiation for Recurrent High-Grade Glioma and Newly Diagnosed Diffuse Midline Glioma/Diffuse Intrinsic Pontine Glioma in Children and Young Adults	GBM6-AD vaccine, topical imiquimod and a single dose of 300cGy reirradiation	Recruiting	I	NCT06305910
Recurrent grade II gliomas	19	A Pilot Study to Evaluate the Effects of Imiquimod and Tumor Lysate Vaccine Immunotherapy for Adults with High Risk or Recurrent/Post-Chemotherapy WHO Grade II Gliomas	BTIC Lysate in combination with imiquimod	Completed	I	NCT01678352
Recurrent glioma	11	A Phase I Study of Vaccination with Lethally Irradiated Glioma Cells Mixed With GM-K562 Cells in Patients Undergoing Craniotomy for Recurrent Tumor	GM-K562 cells mixed with the participants own irradiated tumor cells	Completed	I	NCT00694330
DIPG	8	Imiquimod/BTIC Lysate-Based Vaccine Immunotherapy for Diffuse Intrinsic Pontine Glioma in Children and Young Adults	Imiquimod adjuvant	Terminated	I	NCT01400672
Grade II gliomas	28	Pilot Randomized Neo-adjuvant Evaluation of Poly-ICLC-Assisted Tumor Lysate Vaccines in Adult Patients With WHO Grade II Glioma	GBM6-AD and poly-ICLC before and after surgery; GBM6-AD and poly-ICLC after surgery only	Active, not recruiting	I	NCT02549833
Newly Diagnosed GBM	1	Vaccination of Patients with Newly Diagnosed Glioblastoma Using Autologous Tumor Lysate and Montanide Emulsion for Derivation of Tumor Specific Hybridomas	NA	Terminated	I	NCT01702792
Recurrent HGG	12	Phase I/II Study to Test the Safety and Efficacy of TVI-Brain-1 As A Treatment for Recurrent Grade IV Glioma	Immune adjuvant plused	Completed	I/II	NCT01081223
Recurrent HGG	14	Phase 2 Study to Test the Safety and Efficacy of TVI-Brain-1 As A Treatment for Recurrent Grade IV Glioma	Immune adjuvant and activated white blood cells are plused	Completed	II	NCT01290692

GBM glioblastoma; HGG high-grade glioma; NA not applicable; Clinical trials data collected from https://clinicaltrials.gov/. database.

#### 2.1.2 Peptide vaccines

Peptide vaccines typically consist of 8–31 amino acids and incorporate epitopes that serve as antigenic targets (26). They can be designed using either short peptides, generally 8–10 amino acids in length, or long peptides, which range from 15 to 31 amino acids (22). While earlier clinical trials predominantly employed short peptides, recent studies have increasingly utilized long peptide neoantigen vaccines, as these are capable of eliciting both CD_4_^+^ and CD_8_^+^ T cell responses (27). To enhance immunogenicity, peptide vaccines are frequently conjugated with a carrier protein. However, peptide vaccines are often constrained by HLA/MHC restriction and typically activate a relatively narrow repertoire of antigen-specific T cells, which may limit broad patient applicability and increase vulnerability to immune evasion in heterogeneous gliomas (20). Overall, peptide vaccines exemplify conventional vaccination approaches owing to their structural simplicity that facilitates streamlined manufacturing processes, reduced inter-batch variability, and cost-effective production when compared to other vaccines (22).

#### 2.1.3 Dendritic cell vaccines

DC vaccines represent another investigational vaccine category for glioma therapy (28). The functional role of dendritic cells as professional antigen-presenting cells forms the fundamental basis for DC vaccine mechanisms. Following activation by DCs, immune cells (particularly T lymphocytes) traverse the BBB and enter the brain tumor site to exert antitumor effects (29). Furthermore, DCs are thought to orchestrate dual-phase immune activation, engaging both innate and adaptive immune systems to mediate phenotypic conversion of gliomas from immunologically “cold” to “hot” tumor microenvironments (29–31).

Preloading DCs with the particular tumor antigens prior to immunization can circumvent DC-mediated processing and presentation of tumor antigens (28). Ex vivo-generated autologous DCs are systematically loaded with TAAs via multiple methodologies, including entire tumor lysates, co-culture with viral transfection, antigenic peptides or mRNA electroporation (28). These terminally differentiated, antigen-loaded DCs are subsequently readministered via vaccine formulations, designed to migrate to lymphoid compartments for efficient antigen presentation and priming of antigen-specific T lymphocytes. DC vaccines, through specific targeting of TAAs, potentiate adaptive immune responses capable of mediating sequential recognition, targeting, and elimination of tumor cells (22). To date, multiple DC-based vaccine formulations have been developed and clinically evaluated, targeting both TAAs (e.g., EGFRvIII, WT1, HER2, survivin, IL-13Rα2, MAGE-A3) and tumor-associated neoantigens (e.g., *IDH1R132H*) (32–34).

#### 2.1.4 RNA vaccines

Several key advantages explain why mRNA is preferred for cancer therapeutic vaccines. Notably, mRNA derived from patients can be amplified *in vitro*, enabling the generation of sufficient mRNA from only a small initial cell sample (9). This enables the development of mRNA-based vaccines even when starting with a very small biopsy (35). Additionally, mRNA offers a higher safety profile compared to DNA since there are no risks about integration into the human genome (9, 36). The DC-pulsed mRNA vaccine was one of the first platforms evaluated for mRNA vaccination in human glioblastoma. This strategy can produce a strong CD8+ T cell response, according to preliminary clinical research (37). This induced cytolytic activity is associated with a potential reduction in tumor recurrence and may contribute to extended overall survival in GBM patients (22).

Overall, while these platforms differ in antigen delivery strategy and immunological mechanisms, they share common translational challenges, including limited antigen coverage, immune escape, and variability in clinical efficacy.

## 3 Immunological targets in glioma

The biological rationale of glioma vaccines ultimately depends on the selection of appropriate immunological targets and on whether tumor-specific immune responses can be sustained in the face of immune evasion. Therefore, understanding the antigenic landscape of glioma and its mechanisms of immune resistance is essential for interpreting both vaccine design and clinical performance.

### 3.1 Tumor-associated antigens

TAAs are proteins produced by unmutated genes that are infrequently expressed in normal cells but markedly overexpressed in tumor cells. These overexpressed proteins can be recognized by immune cells, which then trigger the immune system and aid in the destruction of tumor cells. Epidermal growth factor receptor variant III (EGFRvIII) is one of the most well-characterized tumor-associated antigens in glioblastoma, with a reported expression rate of 15%–40% (38). This mutation results from an in-frame deletion of exons 2–7, generating a tumor-specific neoepitope that is absent in normal tissues, making it an attractive target for vaccine-based immunotherapy (39, 40).

Functionally, EGFRvIII is constitutively active and promotes tumor proliferation, invasion, and therapeutic resistance through activation of multiple downstream signaling pathways, including PI3K/Akt and related oncogenic cascades (41–46).

Importantly, while these oncogenic properties support its biological relevance as a therapeutic target, clinical studies have also highlighted key limitations of EGFRvIII-directed approaches, particularly the dynamic loss of antigen expression under therapeutic pressure, which may contribute to immune escape and reduced long-term efficacy. These observations underscore both the rationale and the challenges of targeting single antigens in glioma vaccine development. These considerations also provide the biological rationale for several representative vaccine strategies discussed later in the manuscript.

Beyond EGFRvIII, other glioma-relevant antigens such as survivin, IDH1(R132H), H3K27M, and heat-shock protein-associated peptide complexes have also served as the biological basis for several vaccine platforms discussed later in this review.

### 3.2 Mechanism of immune evasion

Immunotherapeutic approaches have shown biological activity in glioma, although durable efficacy remains limited. Immune checkpoints are surface molecules expressed on immune cells that modulate immune responses through interactions with specific ligands or receptors on tumor cells or other cellular components (47). Tumor cells exploit this regulatory mechanism to evade immune effector cells by expressing checkpoint inhibitors to achieve immune escape (mainly T cells) (48). By attaching to surface receptors on immune cells, checkpoint inhibitors like PD-L1 and CD47, which are extensively expressed on the surface of tumor cells, directly suppress T cell activity and obstruct immunological responses (49), thereby evading the attack of the immune system. Immune checkpoint blockade (ICB) primarily blocks immunosuppressive immune checkpoints including programmed death-1/programmed death-ligand 1 (PD-1/PD-L1) and cytotoxic T-lymphocyte-associated protein 4 (CTLA-4), thus inhibiting their corresponding immunosuppressive effects and exerting an antitumor function. Substantial clinical evidence has demonstrated the therapeutic efficacy of ICB across multiple cancer types (50).

**Figure 3:**
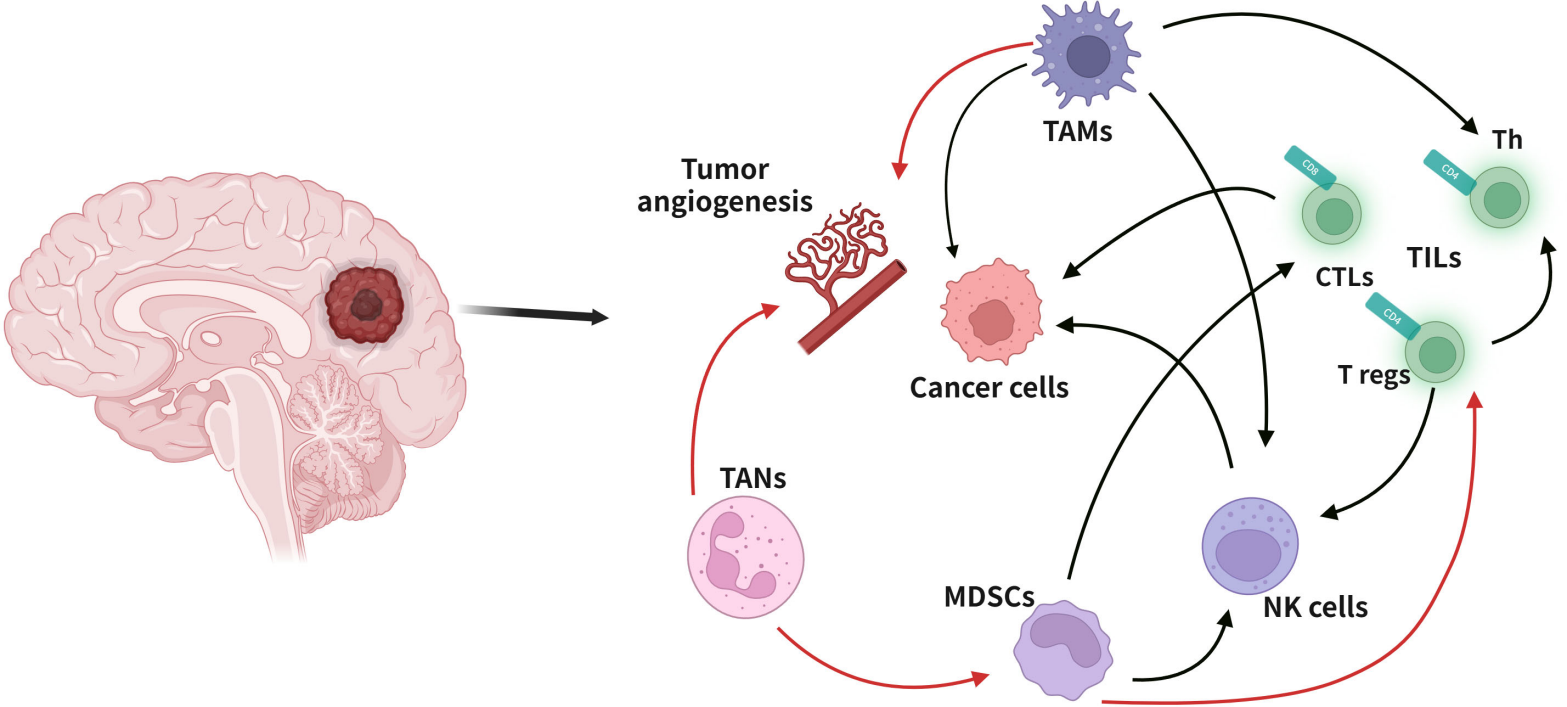
*Cellular composition of glioma immune microenvironment.* The figure depicts only a general representation of all the cell types that have been reported to be associated with tumor cells in glioma immune microenvironment. Black arrow: down-regulation. Red arrow: up-regulation.

However, the unique immunobiology of the brain contributes to a distinct tumor microenvironment (TME) in gliomas, further exacerbating immunosuppression. While the TME contains a variety of peripheral immunological components, such as natural killer cells (NK cells), myeloid derived suppressor cells (MDSCs), CD_4_^+^ helper T cells (Th), CD_8_^+^ cytotoxic T lymphocytes (CTLs), macrophages, neutrophils and regulatory T (T reg) cells (13). However, compared with many other tumor types, the infiltration of these immune cells is unusually limited in gliomas. In addition, a range of cytokines and chemokines derived from tumors reprogram the infiltrating immune cells, leading them to develop distinct functional phenotypes and convert into tumor-associated immune cells. Consequently, these tumor-associated immune cells play a key role in glioma growth, recurrence, and treatment resistance by modulating local inflammatory responses, shifting the balance between pro- and anti-inflammatory signals (6). As conceptually illustrated in **Figure 3**, the glioma microenvironment is not simply immune-poor, but rather immunologically distorted in ways that directly constrain vaccine-induced antitumor immunity. For example, tumor-derived Fas ligand induces apoptosis in activated T cells and facilitates tumor immune escape by concurrently suppressing dendritic cell function and impairing T cell maturation (6).

Tumor-associated macrophages (TAMs), which make up 50% of all immune cells and are the major infiltrating immune population, are particularly significant in the glioma TME (51). Emerging evidence indicates that TAMs promote tumor progression through multiple mechanisms. Firstly, RBPJ-dependent TAMs induce the expansion of PD-1^+^GzmB^-^CD_8_^+^ T cells and inhibit the proliferation of effector T cells, while recruiting Th2 and regulatory T cells, thereby mediating immune suppression. Secondly, TAMs secrete a large number of angiogenic factors such as VEGF and PDGF, promoting tumor angiogenesis. Additionally, TAMs release growth factors and matrix metalloproteinases (MMPs), synergistically enhancing tumor cell proliferation, invasion and metastasis. These immunosuppressive features are not only central to glioma progression but also likely represent major barriers to vaccine efficacy. By limiting antigen presentation, restricting effector T-cell infiltration, and promoting dysfunctional or exhausted immune states, the glioma microenvironment may substantially blunt the translation of vaccine-induced immune priming into durable clinical benefit (51).

## 4 Clinical trials and outcomes

These immunological principles are directly reflected in clinical trial outcomes, where vaccine efficacy is often shaped not only by platform design, but also by antigen selection, tumor evolution, and the immune context in which treatment is delivered.

### 4.1 Overview of clinical trials

The current clinical research landscape encompasses multiple vaccine platforms, including monotherapy and combination therapy approaches, with investigational agents such as peptide-based vaccines (52–60), DC-based vaccines (61–63), nucleic acid vaccines (RNA/DNA) (64), and other autologous vaccine formulations (65) being actively evaluated in ongoing clinical trials which are summarized in **Table 3**. Across these trials, several recurring themes emerge: early-phase studies frequently demonstrate acceptable safety and measurable immunogenicity, whereas durable survival benefit remains inconsistent and appears strongly influenced by antigen selection, tumor heterogeneity, treatment context, and trial design. In view of these shared patterns, the discussion below highlights representative clinical studies not only to summarize efficacy signals, but also to illustrate the mechanistic and translational challenges that have shaped the field. However, the discussion in this review will center specifically on peptide and DC vaccines.

**Table 3:** Key clinical trials investigating therapeutic vaccination against glioma.

Targets	Identifier	Vaccine name	Patient cohort	Phase	Main findings	References
Peptide vaccines						
EGFRvIII	NCT01480479 (ACT IV)	Rindopepimut	745 ndGBM	III	mOS, 20.1 months (95% CI, 18.5-22.1) versus 20.0 months (95% CI, 18.1-21.9) (HR 1.01, 95% CI, 0.79-1.30); mPFS, 8.0 months (95% CI, 7.1-8.5) versus 7.4 months (95% CI, 6.0-8.7) (HR 1.01, 95% CI, 0.80-1.29)	(46)
	NCT01498328 (ReACT)	Rindopepimut	73 rGBM EGFRvIII^+^	II	mPFS, HR 0.72 (95% CI, 0.43-1.21); mOS, HR 0.53 (95% CI, 0.32-0.88)	(47)
Survivin	NCT02455557	SurVaxM	64 ndGBM	IIa	mOS, 25.9 months; mPFS, 11.4 months	(48)
HSP	NCT00293423	HSPPC-96	41 rGBM	II	mOS, 10.7 months (95% CI, 8.7-12.6); 6-month OS, 90.2% (95% CI, 75.9-96.8); 12-month OS, 29.3% (95% CI, 16.6-45.7)	(49)
Personalized antigen/TAAs	NA	PPV HLA-24	88 rGBM	III	mOS, 8.4 months (95% CI, 6.6-10.6) versus 8.0 months (95% CI, 4.8-12.9) (HR 1.13, 95% CI, 0.60-1.90)	(50)
	NCT01222221	IMA952	45 ndGBM receiving IMA950 plus GM-CSF	I	mOS, 15.3 months; 6-month PFS, 74%; 9-month PFS, 31%	(51)
IDH1-R132H	NCT02454634 (NOA-16)	IDH1-vac	33 newly diagnosed IDHmt	I	3-year PFS, 63% (95% CI, 44-77%); 3-year OS, 84% (95% CI, 67-93%)	(52)
H3K27M	NCT04808245	H3-vac	8 H3K27M-mt DMG	I	mOS, 12.8 months; mPFS, 6.2 months	(53)
WT1	NCT01621542	WT2725	44 advanced solid tumors including GBM	I	mPFS, 1.97 months (95% CI, 0.9-11.0); mOS, 10.2 months (95% CI, 4.3-22.5)	(54)
DC vaccines						
TAAs/neoantigens	NCT00045968	DCVax-L	331 GBM;267 ndGBM, 64 rGBM	III	For nGBM, mOS was 19.3 months (95% CI, 17.5-21.3) versus 16.5 months (95% CI, 16.0-17.5) (HR 0.80, 98% CI, 0.00-0.94); For rGBM, mOS 13.2 months (95% CI, 9.7-16.8) versus 7.8 months (95% CI, 7.2-8.2) (HR 0.58, 98% CI, 0.00-0.76)	(55)
	NCT01280552	ICT-107	124 ndGBM	II	mOS, 17.0 months (95% CI, 13.7-20.6) versus 15.0 months (95% CI, 12.3-23.1) (HR 0.87, P = 0.58); mPFS, 11.2 months (95% CI, 8.2-13.1) versus 9.0 months (95% CI, 5.5-10.3) (HR 0.57, P= 0.011)	(56)
	NCT03400917	AV-GBM-1	57 ndGBM	II	mOS, 14.0 months (95% CI, 10.1-18.3); 6-month OS, 87.5%; 1-year OS, 55.4%; 18-month OS, 38.5%; mPFS, 8.5 months (95% CI, 6.5-9.1); 6-month PFS, 69.7%; 1-year PFS, 26.8%; 18-month PFS, 16.1%	(57)
DNA vaccines						
IGF-1R	NCT02507583	IGV-001	33 ndGBM	Ib	mOS, 17.3 months; mPFS, 9.8 months	(58)
Other autologous vaccines						
TAAs	NA	AFTV	24 ndGBM	I/IIa	mOS, 22.2 months; mPFS, 8.2 months; 2-year OS, 47%; 2-year PFS, 33%; 3-year OS, 38%	(59)

mOS median overall survival; mPFS median progression-free survival; Clinical trials data collected from https://clinicaltrials.gov/. database.

#### 4.1.1 Targets (EGFRvIII)

In a randomized, double-blind, phase 3 trial (NCT01480479), 745 patients aged 18 and older with newly diagnosed glioblastoma (ndGBM) expressing EGFRvIII were recruited from 165 hospitals in 22 countries. Patients who met the eligibility requirements had received maximal surgical resection and finished conventional chemoradiation without showing any signs of progression. They were randomly assigned to receive either rindopepimut (371 patients) or a control (374 patients) via monthly intradermal injection, alongside standard oral temozolomide for 6–12 cycles or longer. The primary endpoint was OS in patients with minimal residual disease (MRD). An interim analysis led to the study’s termination for futility. The median overall survival (mOS) of the rindopepimut group and the control group did not differ significantly (20.1 months vs. 20.0 months, HR 1.01, 95% CI, 0.79-1.30; P = 0.93) (52). Common grade 3–4 adverse events included thrombocytopenia, fatigue, brain edema, seizure, and headache. Serious adverse events included seizure and brain edema, with 16 deaths caused by adverse events, one of which was potentially related to rindopepimut.

The double-blind randomized phase II trial (ReACT, NCT01498328) evaluated the efficacy and safety of rindopepimut combined with bevacizumab in patients with relapsed EGFRvIII-expressing glioblastoma. The primary endpoint, 6-month progression-free survival (PFS6), was 28% (10/36) in the rindopepimut group versus 16% (6/37) in the control group (one-sided P = 0.12), meeting the prespecified threshold despite lacking statistical significance. Secondary endpoints demonstrated significant OS improvement in the rindopepimut arm (HR = 0.53, 95% CI, 0.32-0.88, P = 0.01), with 24-month survival rates of 20% vs. 3% in controls (P = 0.0179) (53). Safety analysis revealed primarily grade 1–2 injection-site reactions without serious treatment-related adverse events or discontinuations. This study, the first randomized, placebo-controlled trial in relapsed glioblastoma, demonstrated potential survival benefits of EGFRvIII-targeted immunotherapy combined with antiangiogenic therapy, alongside robust anti-EGFRvIII antibody induction with *in vitro* tumor-lytic activity. In contrast to the negative phase III ACT IV trial in newly diagnosed patients, these results demonstrate a novel immunotherapeutic approach for relapsed glioblastoma despite sample size limitations and bevacizumab response heterogeneity, suggesting bevacizumab may improve vaccine efficacy through immune modulation.

A recurrent challenge in glioma vaccine development is the discrepancy between encouraging early-phase trial results and the neutral or negative outcomes observed in later-stage studies. This pattern is exemplified by rindopepimut, which showed promising immunogenicity and survival signals in phase I/II studies but failed to improve overall survival in the randomized phase III ACT IV trial. Several factors may account for this divergence. First, early-phase trials often enroll highly selected patients with favorable clinical characteristics, such as lower tumor burden, better performance status, and more intact immune competence, which may amplify apparent therapeutic signals. Second, vaccine-induced immunogenicity does not necessarily translate into durable survival benefit, particularly in glioma, where immune activation must overcome profound local and systemic immunosuppression. Third, single-antigen strategies may be especially vulnerable to intratumoral heterogeneity and antigen loss under selective immune pressure, thereby limiting long-term disease control. Finally, larger randomized trials more accurately capture the biological complexity and treatment heterogeneity of glioma in clinical practice, often revealing the limitations of vaccine efficacy that may not be apparent in smaller, earlier-phase studies. Together, these observations suggest that future glioma vaccine development should be guided not only by early immunogenicity signals, but also by platform resilience to antigen escape, biomarker-informed patient selection, and trial designs better aligned with the biological realities of glioma. These findings collectively illustrate a broader challenge in glioma vaccine development, where promising immunogenicity signals frequently fail to translate into durable clinical benefit in larger, more heterogeneous patient populations.

#### 4.1.2 Targets (Survivin)

A multicenter, open-label phase IIa trial (NCT02455557) evaluated the safety and efficacy of SurVaxM, a survivin-targeted peptide vaccine, combined with TMZ in ndGBM. 64 patients were enrolled, with 63 included in the efficacy analysis. The regimen showed encouraging survival outcomes, with a high PFS6 rate and a median overall survival of 25.9 months, while subgroup analyses suggested more favorable outcomes in MGMT promoter-methylated patients and in those mounting stronger humoral responses. Subgroup analyses revealed enhanced survival in MGMT promoter-methylated patients (n=33), achieving an mOS of 41.4 months compared to 16.5 months in unmethylated patients (P<0.01). Patients with anti-SurVaxM immunoglobulin G titers >30,000 (31%) exhibited superior mOS (43.1 months vs. 15.8 months in the low-titer group; HR = 0.41, P = 0.0146) (54). Safety profiles were favorable, with grade 1 injection site reactions (37.5%) as the most common AEs; only two cases of grade 3 localized granulomatous panniculitis were reported, resolving with conservative management. Immunologically, SurVaxM induced survivin-specific CD_8_^+^ T-cell and antibody responses, yet only humoral immunity correlated significantly with survival outcomes. This finding suggests that measurable vaccine-induced immune activation alone may be insufficient unless it is coupled with functionally effective and sustained antitumor immunity within the glioma microenvironment. This study establishes SurVaxM plus TMZ as a promising therapeutic strategy for ndGBM, particularly in MGMT-methylated populations, with manageable toxicity. The synergy between antibody-mediated immunity and chemotherapy underscores a novel mechanism for glioma vaccines. To validate these findings, a phase III randomized experiment is now underway. This pattern further reinforces the notion that quantitative immune activation alone may be insufficient without sustained and functionally effective antitumor immunity within the glioma microenvironment.

#### 4.1.3 Targets (HSP)

Bloch et al (55) conducted a single-arm, phase II study (NCT00293423) to evaluate the efficacy and safety of autologous heat-shock protein peptide complex-96 (HSPPC-96) vaccine for rGBM. 41 patients with gross total resection received postoperative vaccination, and the primary endpoint was OS at 6 months. The study demonstrated a mOS of 42.6 weeks (95% CI, 34.7-50.5) with a promising 6-month OS of 90.2% (95% CI, 75.9-96.8) and a 12-month OS of 29.3% (95% CI, 16.6-45.7) (55, 66). The vaccine exhibited a favorable safety profile, with injection site reactions (41%) as the primary adverse event, only one grade 3 fatigue attributed to vaccination, and no grade 4 events or treatment-related deaths.

#### 4.1.4 Targets (IDH1-R132H)

The pioneering NOA-16 phase I clinical trial (NCT02454634) evaluated the safety and efficacy of IDH1-vac, a vaccine targeting newly diagnosed IDH1(R132H)-mutant gliomas, in 33 patients (32 treated). Results showed vaccine-related adverse events were restricted to Grade 1 (primarily injection-site reactions), with no dose-limiting toxicity. 93.3% (30/32) of patients showed vaccine-induced immune responses, with a 3-year OS rate of 84% (95% CI, 67%-93%) and a 3-year PFS of 63% (95% CI, 44%-77%); patients with immune responses exhibited a two-year PFS of 82%, whereas non-responders progressed within two years (22, 58). Vaccine-induced T cell responses correlated with intratumoral antigen presentation (P = 0.0306). Notably, pseudoprogression (PsPD) occurred in 37.5% of patients, associated with enhanced peripheral T cell responses. Single-cell sequencing revealed clonal expansion of IDH1(R132H)-specific CD_4_^+^ T cells (TCR14) in PsPD lesions. This study pioneers a clonal neoantigen-targeted vaccine for gliomas, circumventing immune evasion from subclonal heterogeneity and eliminating HLA restriction, providing critical evidence for further clinical development. These observations support the concept that vaccines targeting clonally stable neoantigens may be more resistant to immune escape and therefore better positioned to generate biologically meaningful clinical responses than vaccines directed against more heterogeneous or dispensable targets.

#### 4.1.5 Targets (H3K27M)

Grassl et al (59) first reported the compassionate use of an H3K27M-targeted vaccine (H3K27M-vac) in adult patients with progressive H3K27M^+^ diffuse midline glioma (DMG). Among 8 patients, 5 received combined anti-PD-1 therapy. The vaccine demonstrated favorable safety, with only 25% (2/8) experiencing grade 1 injection site reactions and no regimen-limiting toxicity. 62.5% (5/8) of patients had mutation-specific CD_4_^+^ T cell-dominated immunological responses, with mPFS of 6.2 months and mOS of 12.8 months, according to immunogenicity analysis. One patient achieved sustained complete remission >31 months after pseudoprogression. This study provides a novel strategy for vaccine development in low-mutational-burden brain tumors, supporting further efficacy validation in newly diagnosed patients combined with standard therapies.

#### 4.1.6 DC vaccines

Most dendritic cell vaccine studies in glioma remain in the early clinical stage (phase I/II); however, one of the most clinically advanced and widely discussed studies is the phase III DCVax-L trial (NCT00045968), an autologous tumor lysate-loaded dendritic cell vaccine (DCVax-L) was evaluated alongside the standard of care (SOC) for both ndGBM and rGBM, compared to a contemporaneously matched external cohort (61). At first, 331 patients with ndGBM were enrolled after SOC, with 232 and 99 receiving DCVax-L and placebo, respectively. Nevertheless, 120 patients who were initially assigned to the DCVax-L group maintained the treatment, whereas 64 patients from the placebo group who had a recurrence were later switched over to receive DCVax-L. Patients getting standard treatment or other approved therapies made up an external control cohort. Among the 232 newly diagnosed and 64 recurrent GBM patients treated with DCVax−L, median OS was significantly extended compared with the matched external cohort (22.4 vs. 16.5 months for newly diagnosed GBM; 13.2 vs. 7.8 months for recurrent GBM) (61). Although DCVax-L has shown a survival benefit in both ndGBM and rGBM, important methodological concerns remain, particularly regarding the externally controlled comparator and crossover design, both of which complicate interpretation of the reported survival benefit and may limit generalizability. While this DCVax-L trial reported encouraging survival extensions, the vaccine also has limitations such as the need for individualized preparation and high costs. Future Phase III trials must prioritize rigorous, randomized, placebo-controlled designs to definitively validate these clinical benefits (22). This example highlights how trial design factors, including control selection and crossover, can significantly influence the interpretation of vaccine efficacy in glioma.

#### 4.1.7 DNA vaccines

IGV-001, a vaccine that combines autologous glioblastoma cells with an IGF-1R antisense oligonucleotide (IMV-001), was tested for safety and effectiveness in ndGBM patients in a phase Ib clinical trial (NCT02507583) (64). Results showed a mPFS of 9.8 months in the intent-to-treat (ITT) group with 33 recruited patients and a median follow-up of 3.1 years, which was considerably better than the historical SOC of 6.5 months (P = 0.0003). In Stupp-eligible subgroups (n=22), mPFS was 11.6 months (P = 0.001), while the highest-exposure cohort (20 biodiffusion chambers, 48-hour exposure, Cohort D; n=10) achieved median PFS and mOS of 17.1 months (P = 0.0025) and 38.2 months (P = 0.044), respectively. Patients with methylated MGMT promoters (n=10) showed a mPFS of 38.4 months (P = 0.0008). According to safety statistics, there were no severe immune-related toxicities and six individuals had grade ≤ 3 treatment-related side events, suggesting favorable tolerability. Compared to checkpoint inhibitors and chimeric antigen receptor T-cell (CAR-T) therapies, IGV-001 exhibited superior safety, offering breakthrough insights for glioma vaccine development.

In addition, interpretation of vaccine efficacy in glioma should account for treatment-related immune constraints, particularly corticosteroid exposure and therapy-induced lymphopenia, both of which may attenuate vaccine-primed antitumor responses in clinical settings. Across these clinical studies, several recurring factors appear to influence therapeutic outcomes, including antigen heterogeneity within gliomas, immune escape mechanisms following selective pressure, HLA restriction in peptide-based vaccines, the immunosuppressive effects of corticosteroid use, and variability in trial design and patient selection. These factors should be considered when interpreting the efficacy signals reported across different vaccine platforms.

Taken together, these studies suggest that the limited and heterogeneous clinical efficacy of glioma vaccines is not attributable to a single factor, but rather reflects the combined effects of antigen heterogeneity, treatment context, immune suppression, and trial design limitations.

## 5 Immunotherapy combinations

### 5.1 Rationale for combination therapy

**Figure 4:**
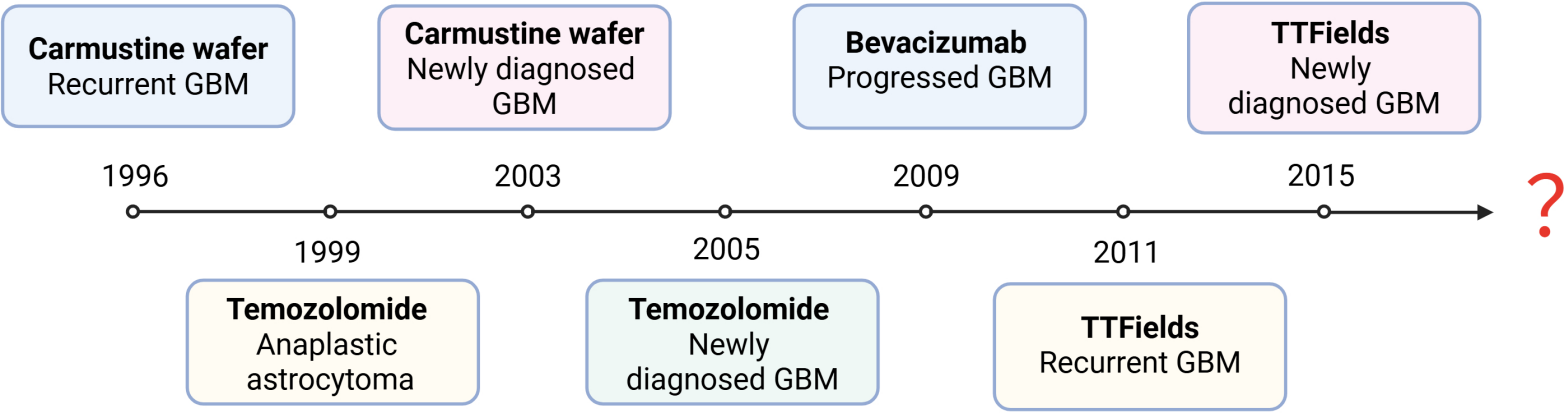
Evolution of FDA-approved GBM treatment approaches.

GBM is widely acknowledged as a profoundly aggressive primary brain tumor, defined by marked intratumoral heterogeneity, an immunosuppressive TME, and a predilection for recurrence (67, 68). This pathological complexity has made combination therapies a clinical necessity, an approach neurosurgeons adopted early in clinical practice. A classic example is the Stupp protocol, which combines maximal safe resection with concomitant radiochemotherapy (10). More recently, the introduction of novel therapeutic modalities, including immunotherapy, targeted agents, and tumor-treating fields, has enabled the design of modern combination regimens that show considerable promise in both preclinical and clinical studies (69). As outlined in **Figure 4**, this therapeutic evolution provides the broader clinical context in which vaccine-based approaches are increasingly being integrated into multimodal glioma treatment strategies.

In the context of glioma vaccines, combination therapy can be conceptualized as a strategy to overcome the sequential barriers that limit durable antitumor immunity. Vaccines primarily function as immune-priming platforms by initiating tumor-specific T-cell and/or humoral responses. However, effective tumor control requires additional processes beyond priming, including sufficient antigen release, immune-cell trafficking into the tumor bed, reversal of local immunosuppression, and sustained effector activity under selective tumor pressure.

This is particularly relevant in glioma, where vaccine-induced immune priming alone may be insufficient to overcome the profoundly immunosuppressive tumor microenvironment. Accordingly, rational combinations aim not only to enhance antigen-specific immune activation but also to remodel the local immune milieu, improve T-cell infiltration, and reverse adaptive resistance mechanisms. Immunotherapy works by reprogramming and utilizing the patient’s immune system to fight tumors by inducing immunogenic cell death, which is mostly caused by apoptosis, necrosis, or autophagy (5, 24, 47). Contemporary immunotherapeutic strategies in cancer treatment predominantly focus on immune checkpoint inhibitors (ICIs) (24). Combination approaches in glioma are increasingly being explored because they may help overcome the limitations of monotherapy through synergistic integration of ICIs with adjuvant radiotherapy, chemotherapy, or TMZ. Such approaches may help mitigate the inherent limitations of singular treatment modalities by addressing two critical challenges, suboptimal eradication of residual tumor cells following surgical resection, and non-selective cytotoxicity associated with traditional chemo/radiotherapy (70). Mechanistically, immunomodulatory interventions during therapeutic intervals enhance antitumor immunity through selective amplification of immune effector functions, while simultaneously counteracting treatment-induced immunosuppression (71). These combinatorial strategies may offer dual clinical advantages by enhancing tolerance to cytotoxic therapies’ adverse effects and substantially improving long-term outcomes through reduced recurrence rates and metastasis prevention, ultimately establishing a paradigm shift in neuro-oncological management.

### 5.2 Strategic combinations and synergistic therapies

Cancer immunotherapy refers to therapeutic strategies that harness patients’ own immune systems for cancer recognition and elimination (72). Current immunotherapeutic approaches for gliomas primarily comprise four categories: ICBs, vaccination strategies, CAR-T therapies, and oncolytic viruses (OVs) (73, 74). ICB therapy functions by antagonizing immune checkpoints such as PD-1/PD-L1, thereby counteracting immunosuppressive signaling. Peptide and dendritic cell vaccines utilize TAAs and tumor-specific antigens (TSAs) as immunological targets. CAR-T cell therapies engineered to target tumor surface molecules including EGFRvIII, interleukin-13 receptor α2 (IL-13Rα2), and human epidermal growth factor receptor 2 (HER2) have demonstrated investigational potential in GBM management (75–77). OV-based interventions represent a novel therapeutic modality for GBM that has garnered significant research interest, exemplified by the conditional regulatory approval of third-generation oncolytic HSV-1 variant G47Δ in Japan, paving the way for advancements in neuro-oncological immunotherapy (78). The major categories of combination strategies can be broadly interpreted according to their dominant biological function: enhancing antigen release and immune priming, reversing immunosuppressive signaling, improving immune access to the tumor bed, or counteracting adaptive resistance. Representative combination strategies discussed in this section are summarized in **Table 4**.

**Table 4:** Representative combination immunotherapy strategies in glioma.

Combination strategy	Representative trial	Biological rationale	Key clinical findings
Vaccine + Standard of Care (SOC)	DCVax-L (NCT00045968)	Surgery and chemoradiotherapy reduce tumor burden and may enhance antigen release, while vaccination promotes tumor-specific immune priming	Improved OS signal in ndGBM and rGBM compared with external controls, though interpretation is limited by crossover and externally controlled design
Vaccine + Temozolomide (TMZ)	SurVaxM (NCT02455557)	Vaccine-induced immune priming may help sustain tumor-specific immune surveillance during the adjuvant TMZ phase following chemoradiotherapy	Favorable safety, strong immunogenicity, and encouraging survival outcomes in ndGBM
Vaccine + Anti-angiogenic Therapy	ReACT (NCT01498328)	Anti-VEGF therapy may improve immune access and reduce VEGF-mediated immunosuppression, potentially enhancing vaccine efficacy	Improved OS signal and steroid reduction, though primary endpoint benefit was modest
Immune Checkpoint Inhibitor (ICI) + Anti-angiogenic Therapy	Pembrolizumab ± bevacizumab (NCT02337491); CheckMate 143 (NCT02017717)	Combines reversal of immune checkpoint suppression with vascular normalization and modulation of the tumor microenvironment	Limited overall clinical benefit despite some short-term disease control and subgroup signals
ICI + CAR-T Therapy	CAR T-EGFRvIII + pembrolizumab (NCT03726515)	Checkpoint blockade may mitigate T-cell exhaustion and support CAR-T persistence and function	Biologically active but no clear survival improvement; resistance mechanisms included antigen loss and T-cell dysfunction
ICI + Oncolytic Virus (OV)	DNX-2401 + pembrolizumab (NCT02798406)	OVs promote immunogenic tumor cell death and inflammatory remodeling of the TME, which may synergize with checkpoint blockade	Favorable safety, durable responses in selected patients, and encouraging 12-month OS

#### 5.2.1 Vaccines combined with SOC

The combination of DCVax-L, an autologous dendritic cell vaccine, with SOC therapy for GBM is mechanistically grounded in activating tumor-specific T-cell immunity through antigen-presenting dendritic cells while leveraging SOC modalities like surgery and chemoradiation to reduce tumor burden and enhance immunogenicity. In the pivotal phase III trial (NCT00045968, N = 331), the DCVax-L was investigated in combination with SOC therapy for patients with ndGBM and rGBM. Clinically, ndGBM patients receiving DCVax-L exhibited significantly extended mOS of 19.3 months (vs 16.5 months in controls) with 48-/60-month survival rates of 15.7%/13.0% (vs 9.9%/5.7%), while rGBM patients demonstrated a mOS of 13.2 months (vs 7.8 months) and 24-/30-month survival rates of 20.7%/11.1% (vs 9.6%/5.1%), with MGMT-methylated ndGBM subgroups achieving particularly favorable outcomes (21.3-month mOS vs external controls, p=0.03) (61). Notably, this phase III study represents one of the most clinically advanced evaluations of vaccine-based therapy in glioma; however, interpretation of its efficacy remains influenced by the use of external controls and crossover design. Individual patient-level information from outside control groups is still unavailable, though. Furthermore, to thoroughly assess the clinical effectiveness of DCVax-L in the treatment of GBM, rigorous prospective investigations, such as randomized controlled trials, are necessary (69).

#### 5.2.2 Vaccines combined with TMZ

SurVaxM, a survivin-targeted peptide vaccine, operates on the biological principle of eliciting survivin-specific immune responses, highlighted by CD_8_^+^ T-cell activation and robust antibody generation, which are instrumental in extending survival outcomes. In preclinical tumor models, the synthetic survivin peptide vaccine SurVaxM has shown substantial antitumor activity (79, 80). Subsequently, an initial clinical trial in patients with recurrent malignant glioma following standard treatment confirmed its favorable safety and tolerability profile. Notably, seven of eight patients achieved survival exceeding one-year post-treatment initiation (81). Thus, Manmeet et al. (54) evaluated the safety, immunologic effects, and survival outcomes of SurVaxM (a survivin-targeted peptide vaccine) combined with adjuvant TMZ in ndGBM. In this open-label, multicenter phase IIa trial, 64 patients with resected ndGBM were treated with a four-dose priming regimen of SurVaxM. Each dose consisted of 500 μg administered every two weeks, formulated with Montanide ISA-51 and sargramostim. Following the priming phase, patients received adjuvant TMZ alongside maintenance doses of SurVaxM, continuing this combined treatment until disease progression (54). Results demonstrated favorable tolerability with no AEs attributed to SurVaxM. Among 63 evaluable patients, 95.2% remained progression-free at 6 months post-diagnosis (prespecified primary endpoint), with mPFS and mOS of 11.4 months (95% CI, 9.9-12.7) and 25.9 months (95% CI, 22.5-29.0) from the first vaccine dose. Immunologic analyses revealed survivin-specific CD_8_^+^ T-cell activation and antibody responses (80% achieved titers≥1:10,000), with higher titers (>30,000) significantly correlating with improved OS (P = 0.0146). *Post hoc* analysis indicated survival benefit from SurVaxM treatment in both methylation subgroups. Patients with methylated MGMT promoter status achieved a mOS of 41.4 months (95% CI, 32.1-49.4), while those with unmethylated status had an mOS of 16.5 months (95% CI, 13.4-19.3). The strong PFS-OS correlation (r=0.79) supported PFS as a potential surrogate endpoint. These findings support the potential of SurVaxM plus TMZ as a clinically promising strategy; however, the absence of a randomized control arm and the relatively small sample size warrant cautious interpretation pending confirmation in ongoing placebo-controlled trials (54).

#### 5.2.3 Vaccines combined with targeted therapy

The combination of rindopepimut, an EGFRvIII-targeted peptide vaccine, with bevacizumab, an anti-VEGF monoclonal antibody, was biologically rationalized by leveraging rindopepimut’s induction of tumor-specific humoral immunity against the constitutively active EGFRvIII oncoprotein, coupled with bevacizumab’s anti-angiogenic effects and potential immune modulation in the tumor microenvironment. In this phase II, double-blind trial (ReACT; NCT01498328), 73 bevacizumab-naïve patients with relapsed EGFRvIII-positive glioblastoma were assigned 1:1 to receive rindopepimut plus bevacizumab or control (KLH plus bevacizumab), with the primary endpoint of PFS6 assessed by central review (53). The rindopepimut arm demonstrated a higher PFS6 rate (28% vs. 16%; P = 0.12), significantly improved overall survival (95% CI 0.32-0.88), higher objective response rate (30% vs. 18%), and greater steroid reduction (33% vs. 0% discontinuation ≥6 months). Safety profiles were comparable, with primarily low-grade injection site reactions. Key limitations include small sample size, potential imbalances in baseline prognostic factors (e.g., prior surgeries, relapse status), and reliance on archival tumor samples for EGFRvIII assessment, which may underestimate antigen loss at recurrence. Validation in a larger phase III trial with centralized biomarker verification is warranted. While the study signals potential synergy between targeted immunotherapy and anti-angiogenic therapy, the modest primary endpoint improvement and historical failure of rindopepimut in newly diagnosed settings (ACT IV) underscore the need for cautious interpretation of phase II efficacy signals in this heterogenous population.

The divergent outcomes between the ACT IV (ndGBM) and ReACT (rGBM) trials offer critical insights into trial design and resistance mechanisms. The failure of the ACT IV trial suggests that targeting a single epitope (EGFRvIII) as a monotherapy is insufficient for newly diagnosed patients due to antigenic drift and the emergence of EGFRvIII-negative clones. In contrast, the promising signals in the ReACT trial may be attributed to the synergistic effect of bevacizumab. Bevacizumab not only controls peritumoral edema but also potentially modulates the immunosuppressive microenvironment by reducing VEGF-mediated inhibition of T-cell infiltration, thereby enhancing the delivery and efficacy of vaccine-induced antibodies.

#### 5.2.4 ICIs combined with targeted therapy

In the largest phase 3 randomized trial of an immune checkpoint inhibitor in recurrent GBM reported to date (CheckMate 143, NCT02017717), Reardon and colleagues aimed to assess whether the PD-1 inhibitor nivolumab improved survival compared with bevacizumab in this patient population (66, 82). The biological rationale for this combination therapy is based on targeting different mechanisms of tumor progression, where ICIs like nivolumab and pembrolizumab unleash the immune system by inhibiting the PD-1/PD-L1 pathway while traditional agents like bevacizumab reduce tumor vascularization through VEGF inhibition, possibly enhancing immune cell infiltration. The study randomized 369 patients with first recurrence to nivolumab (n=184) or bevacizumab (n=185). The results showed no statistically significant difference in mOS between the two groups. The nivolumab arm had an mOS of 9.8 months, compared to 10.0 months for the bevacizumab arm (hazard ratio = 1.04; P = 0.76). The 12-month OS rate was identical at 42% in both. Bevacizumab demonstrated a higher objective response rate (ORR) (23.1% vs 7.8%), but nivolumab responses were more durable (median 11.1 vs 5.3 months). Grade 3/4 treatment-related adverse events were comparable (18.1% vs 15.2%). Exploratory subgroup analysis suggested potential OS benefit with nivolumab in patients with methylated MGMT promoter and no baseline corticosteroid use (mOS 17.0 vs 10.1 months with bevacizumab) (82). These results collectively demonstrate that nivolumab showed comparable survival and consistent safety to bevacizumab in rGBM, while a subset (MGMT-methylated, corticosteroid-free) might benefit from ICIs, warranting further investigation into combination therapies. Also, it is important to acknowledge that the transition of these combination strategies into clinical success has been met with modest outcomes. For instance, large-scale Phase III trials such as CheckMate 143, 498, and 548, which explored PD-1 inhibition in various GBM settings, failed to demonstrate a significant overall survival benefit compared to standard care (83).

Nayak et al. (84) conducted a multicenter, two-cohort randomized phase II research (NCT02337491) to evaluate the efficacy of pembrolizumab (anti-PD-1 therapy) combined with bevacizumab (anti-VEGF therapy) versus pembrolizumab monotherapy in patients with rGBM. The trial enrolled 80 bevacizumab-naive rGBM patients, randomized to cohort A (50 patients, pembrolizumab + bevacizumab) or cohort B (30 patients, pembrolizumab alone), with the primary endpoint of PFS6. Results revealed PFS6 rates of 26.0% (95% CI, 16.3-41.5) and mOS of 8.8 months (95% CI, 7.7-14.2) for cohort A, with an ORR of 20%; cohort B had a PFS6 of 6.7% (95% CI, 1.7-25.4), mOS of 10.3 months (95% CI, 8.5-12.5), and ORR of 0%. Overall, these findings suggest that although combination therapy may improve short-term disease control in some patients, its overall clinical benefit remains limited.

#### 5.2.5 ICIs combined with CAR-T

The combination of CAR T-EGFRvIII cells and pembrolizumab is based on the rationale that enhancing antitumor immune response, involving engineered T cells targeting EGFRvIII-positive glioblastoma along with an immune checkpoint inhibitor to mitigate immune suppression. Bagley et al. (85) undertook a phase I trial (NCT03726515) to assess the safety and tolerability of repeated peripheral infusions of CAR T-EGFRvIII cells combined with pembrolizumab in seven patients with newly diagnosed EGFRvIII-positive glioblastoma. Results showed the combination appeared to be well tolerated with no dose-limiting toxicities (DLTs), but mPFS was 5.2 months (90% CI, 2.9-6.0 months) and mOS was 11.8 months (90% CI, 9.2-14.2 months), demonstrating no improvement over historical controls.

Despite administration of up to three CAR T-cell infusions, peak peripheral engraftment levels remained nearly a log lower than those observed in rGBM patients receiving a single dose of EGFRvIII-targeted CAR T cells, and post-treatment tumors exhibited increased T-cell exhaustion markers (e.g., TOX, PDCD1, PD-1) and regulatory T cells, with CAR T cells detected in only one patient’s tumor (85). Exploratory analyses revealed elevated interferon (IFN)-stimulated T cells in the TME post-treatment, correlating with survival, yet reduced EGFRvIII expression (6/7 patients) failed to translate to clinical benefit. The study concludes that while the combination is safe and biologically active, its lack of efficacy necessitates alternative strategies to address tumor heterogeneity, insufficient CAR T-cell expansion, and the immunosuppressive TME barriers (86, 87). Together, these findings illustrate how adaptive resistance, antigen downregulation, and dysfunctional T-cell states can blunt clinical efficacy despite evidence of biological activity.

#### 5.2.6 ICIs combined with OVs

The antitumor effects of OVs are principally mediated through two distinct mechanisms: direct cytolytic destruction of malignant cells and modulation of the immunosuppressive tumor microenvironment (88). Immune-mediated antitumor responses, induced by OVs and augmented with checkpoint inhibition, could represent an effective therapeutic strategy for GBM. Nassiri et al. (89) aimed to evaluate the safety and efficacy of OVs DNX-2401 combined with anti-PD-1 antibody pembrolizumab in rGBM. A multicenter phase 1/2 trial (NCT02798406) enrolled 49 patients receiving intratumoral DNX-2401 followed by intravenous pembrolizumab, divided into dose-escalation and dose-expansion phases. The results indicated that the combination therapy exhibited a favorable safety profile, with no DLTs observed, meeting the primary safety endpoint. The primary efficacy endpoint of ORR was 10.4% (90% CI, 4.2-20.7%), not statistically superior to the prespecified 5% threshold, while the secondary endpoint of 12-month OS significantly improved to 52.7% (95% CI, 40.1-69.2%), statistically surpassing the prespecified control rate of 20% (89). Besides, three patients finished their treatment with long-lasting full responses that lasted longer than 45 months, and gene expression analyses revealed that patients with TME^median^ tumors showed enhanced clinical benefit (95% CI, 1.02-19.4) (90). These findings suggest that this combination may confer benefit in selected patients and highlight tumor microenvironment features as potential biomarkers; however, the modest objective response rate and the early-phase nature of the study indicate that further validation is still required before broader clinical conclusions can be drawn.

### 5.3 Clinical bottlenecks and potential solutions

Despite advancements in combinatorial immunotherapies for glioblastoma, several challenges persist. First, antigen heterogeneity, exemplified by EGFRvIII loss in recurrent tumors post-CAR-T therapy, limits durable responses, necessitating multi-targeted approaches or real-time biomarker monitoring to address clonal evolution. Second, immunosuppressive TME, characterized by T-cell exhaustion and regulatory T-cell infiltration, remains a critical barrier. Strategies combining TME-modulating agents (e.g., OVs with ICIs) or targeting specific inhibitory pathways (e.g., VEGF/PD-1 dual blockade) show promise in preclinical and early-phase trials. Furthermore, delivery and dosage, such as insufficient CAR-T cell engraftment or restricted intratumoral viral distribution, underscores the need for enhanced delivery systems or optimized dosing schedules to improve therapeutic penetration. Lastly, trial design limitations, including small sample sizes, reliance on historical controls, and heterogeneous patient populations, highlight the imperative for large-scale randomized trials with centralized biomarker validation and stratified subgroup analyses.

Vaccines’ potential to produce significant therapeutic and clinical effects in glioma immunotherapy is based on their functional mechanisms. Future studies should therefore focus on a deeper investigation into the distinct ways these vaccines engage and modulate anti-glioma immune responses (1). Advancing our understanding of vaccine mechanisms and their combination with other immunotherapies will help elucidate key immune and biological processes in glioma, thereby informing more effective research and treatment strategies. Furthermore, additional preclinical and clinical trials are necessary to comprehensively assess possible synergistic effects when paired with existing therapy methods, given the non-negligible roles of vaccinations (1). A structured overview of the major combinational strategies, their biological rationale, and current limitations is summarized in **Table 5**.

**Table 5:** Issues with clinical bottlenecks and potential solutions.

Clinical bottlenecks	Potential solutions
Antigen heterogeneity	Real-time biomarker monitoring
Immunosuppressive TME	Combination of TME-modulating agents or targeting specific inhibitory pathways
Delivery and dosage	Enhancing delivery systems or optimizing dosing schedules
Trial design limitations	Conduct large-scale randomized trials

## 6 Future directions

Beyond incremental platform optimization, the development of next-generation glioma vaccines will likely depend on several broader design principles derived from the translational limitations of current approaches. First, future vaccine platforms should prioritize stable and biologically meaningful antigens, particularly clonal neoantigens or stemness-associated targets, to reduce the risk of antigen escape and improve long-term immune pressure. Second, vaccine development should move beyond immunogenicity alone as a surrogate of success, and instead emphasize the durability, functional quality, and clinical relevance of the induced immune response. Third, vaccine strategies should be co-designed with the tumor microenvironment in mind, as effective antitumor immunity in glioma will likely require simultaneous reversal of local immunosuppression and support for immune-cell infiltration and persistence. Finally, biomarker-guided development pipelines integrating multi-omics profiling, single-cell analysis, and patient-specific modeling may be essential for selecting appropriate targets, stratifying patients, and improving translational predictability.

Building upon these principles, several practical directions may further advance the development of glioma vaccines. To promote the development of novel vaccine types, a meticulous selection of tumor-specific neoepitopes or neoantigens should be given priority, with special attention paid to their association with tumor stemness and antigen persistence. Furthermore, substantial efforts should be directed toward establishing individual-mimic preclinical models that accurately recapitulate individual tumor biology. Ideally, such models should incorporate patient-derived tumor organoids or xenografts together with autologous or reconstructed immune components, thereby enabling more physiologically relevant evaluation of antigen presentation, immune-cell infiltration, and treatment response under patient-specific tumor–immune conditions. In parallel, multi-omics profiling should be embedded prospectively into clinical trial design rather than applied only retrospectively. This may include baseline genomic and transcriptomic profiling for neoantigen prioritization, longitudinal single-cell and TCR sequencing to monitor immune dynamics during treatment, and spatial transcriptomics to characterize intratumoral immune heterogeneity and treatment-induced remodeling. Such integrated strategies may facilitate real-time biomarker discovery, patient stratification, and adaptive immunotherapy trial design. These approaches may further support the identification of multimodal biomarkers predictive of vaccine responsiveness and resistance. These approaches may support biomarker discovery, patient stratification, and adaptive trial design. The development of DNA vaccine vectors to deliver antigen-specific CARs or TCRs to improve therapeutic efficacy is another important topic. Given the encouraging preliminary results from early-phase clinical trials employing novel CAR-T modalities, there exists an imperative need for systematic evaluation of vaccine monotherapy versus combination strategies with adoptive cell transfer (ACT) or ICIs through rigorously designed large-scale clinical investigations with standardized protocols (91, 92). Finally, experimental designs must align with the new glioma classification criteria established in recent diagnostic guidelines. Collectively, while requiring further refinement, glioma vaccines represent promising immunotherapeutic modalities that hold strong potential to provide clinical benefit for glioma patients (22).

## 7 Conclusion

Glioma vaccines have emerged as one of the most conceptually compelling immunotherapeutic strategies in neuro-oncology, particularly because they offer the possibility of inducing tumor-specific and durable immune responses in a disease with otherwise limited treatment options. Taken together, the evidence reviewed in this work suggests that several unifying principles may govern the success or failure of glioma vaccine strategies. First, effective vaccine design must account for antigen stability and intratumoral heterogeneity, as single-target approaches remain highly vulnerable to immune escape. Second, the glioma tumor microenvironment represents a dominant barrier to therapeutic efficacy, requiring combination strategies that simultaneously promote immune activation and relieve local immunosuppression. Third, clinical translation depends not only on immunogenicity but also on the durability and functional quality of the induced immune response, highlighting the importance of biomarker-guided patient selection and rational trial design. However, despite substantial progress in antigen discovery, platform development, and early-phase clinical testing, their clinical translation has remained inconsistent. A central challenge is that vaccine-induced immunogenicity does not reliably translate into durable therapeutic benefit in glioma. This disconnect reflects several unresolved barriers, including intratumoral antigen heterogeneity, adaptive immune escape, HLA restriction in selected platforms, treatment-related immunosuppression, and the profoundly inhibitory tumor microenvironment. In parallel, current clinical trial designs have often been insufficiently aligned with the biological complexity of glioma, limiting the interpretation and generalizability of encouraging early-phase signals.

Moving forward, meaningful progress in this field will likely depend on a shift from isolated vaccine development toward biologically integrated therapeutic design. This includes improved antigen prioritization, incorporation of multi-antigen or personalized neoantigen strategies, biomarker-guided patient selection, and rational combination approaches aimed at overcoming microenvironmental resistance. Equally important will be the development of more predictive translational models and clinically relevant endpoints capable of better capturing immunotherapeutic activity.

Overall, glioma vaccines should no longer be viewed simply as standalone experimental interventions, but rather as potential components of a broader, mechanism-informed immunotherapeutic framework. Their future success will depend on how effectively vaccine design can be integrated with tumor biology, immune context, and clinical strategy.
